# Comparison of the short-term outcomes of using DST and PPH staplers in the treatment of grade III and IV hemorrhoids

**DOI:** 10.1038/s41598-020-62141-5

**Published:** 2020-03-23

**Authors:** Tzu-Hsuan Wang, Kee-Thai Kiu, Min-Hsuan Yen, Tung-Cheng Chang

**Affiliations:** 1 Division of colorectal surgery, Department of surgery, Taipei Medical University Shuang-Ho Hospital, Taipei City, Taiwan; 2 Department of surgery, school of medicine, college of medicine, Taipei Medical University, Taipei City, Taiwan

**Keywords:** Anus, Anal diseases, Anal diseases

## Abstract

Stapled hemorrhoidopexy has a few advantages such as less postoperative pain and faster recovery compared with conventional hemorrhoidectomy. There are two major devices used for stapled hemorrhoidopexy, PPH stapler (Ethicon EndoSurgery) and DST stapler (Covidien). This study was conducted to investigate the postoperative outcomes among patients with grade III and IV hemorrhoids who underwent hemorrhoidopexy with either of these two devices. A total of 242 consecutive patients underwent stapled hemorrhoidopexy with either PPH stapler (110 patients) or DST stapler (132 patients) at a single center in 2017. We performed a retrospective case–control study to compare the short-term postoperative outcomes and the complications between these two groups. After matching the cases in terms of age, gender, and the grade of hemorrhoids, there were 100 patients in each group (PPH versus DST). There were no significant differences in the postoperative visual analog scale (VAS) score and analgesic usage. Among complications, the incidence of anorectal stricture was significantly higher in the DST group (*p* = 0.02). Evaluation of the mucosal specimen showed that the total surface area, the muscle/mucosa ratio and the surface area of the muscle were also significantly higher in the DST group (*p* = 0.03). Further analysis of the DST group demonstrated that patients with anorectal stricture after surgery are younger than patients without anorectal stricture, and higher muscle/mucosa ratio (*p* = 0.03) and a higher surface area of the muscle (*p* = 0.03) also measured in the surgical specimen. The two devices provide similar outcomes of postoperative recovery. Patients who underwent DST stapled hemorrhoidopexy had a higher incidence rate of stricture, larger area of muscle excision, and higher muscle/mucosa ratio in the surgical specimen. Further investigation is warranted for a better understanding of the correlation between muscle excision and anorectal stricture.

## Introduction

The hemorrhoidal disease affects approximately 4.4–36%^1,2^ of the general population, and it has been estimated that >50% of the population aged >50 years experiences hemorrhoidal problems^3^. Traditional hemorrhoidectomy, including Milligan–Morgan^4^, Ferguson^5^, and Whitehead procedures^6^, are known to cause significant postoperative pain and discomfort and result in a relatively better but unsatisfying quality of life after operation^7^. Since the first introduction of the novel procedure hemorrhoidopexy by Longo in 1998^8^, it has been considered as a safe procedure causing less postoperative pain and resulting in earlier recovery^9–13^. The PPH stapler (Ethicon Endo-Surgery, Inc. Cincinnati, OH, USA) was also first introduced in 1998 as a device to perform this procedure. Subsequently, a new device, the DST stapler (Covidien, Mansfield, MA, USA), was introduced in 2008 with some structural differences, including a detachable anvil, three anchor points over different levels, a larger case, and different staple sizes.

However, the majority of current studies have been focusing on the use of PPH stapler for hemorrhoidopexy, and comparison with the DST stapler has been rarely discussed. One randomized controlled trial that compared between the PPH stapler and the DST stapler reported that the DST stapler demonstrated a better hemostatic ability and allowed the resection of a larger area of mucosal prolapse^14^. However, that trial focused only on bleeding among the postoperative complications. Currently, only a limited number of studies have compared these two devices in terms of pain, complications, and anorectal stricture incidence rate. The present investigation is a matched cohort–control study aimed to compare the postoperative short-term outcomes among patients with grade III and IV hemorrhoids that were received stapled hemorrhoidopexy (SH) with either the PPH or the DST stapler. The specimen surface area and the relationships with complications were also analyzed.

## Patients and methods

**Figure 1:**
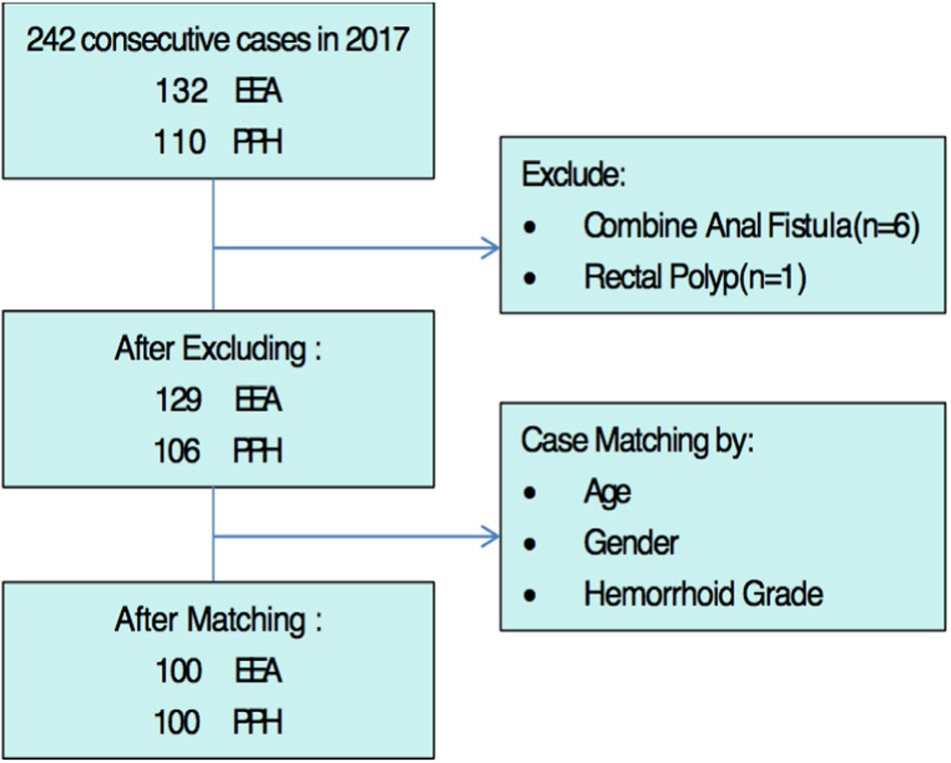
The algorithm in each groups.

A total of 242 consecutive patients who underwent SH from January to December 2017 were enrolled in this study. All of patients had grade III and IV hemorrhoids. Patients with anal fistula and rectal polyp were excluded, and cohort matching in terms of age, gender, grade of hemorrhoidal disease was done, due to these factors might significantly affect hemorrhoids characteristics then causing selection bias. Finally, there were 100 patients in each group. The PPH group of patients underwent SH using the PPH03 stapler (Ethicon Endo-Surgery, Inc. Cincinnati, OH, USA), whereas patients in the DST group underwent SH using the DST stapler (3.5 mm) (Covidien, Mansfield, MA, USA). The selection between the two devices was dependent on the storage supply; there was a temporary shortage of PPH staplers in the first half year and a shortage of DST staplers in the second half year at Shuang-Ho Hospital. We introduced these two devices to our institution since 2015, and have already treated more than 200 patients by each device before the study started. In this study, all surgeries were performed by a single experienced colorectal surgeon who evaluated the patients and made surgical decisions using the same standards. The algorithm showing patient selection is depicted in Fig. 1. This study was conducted according to guidelines laid down in declaration of Helsinki and all procedures involving participants were approved by the Taipei Medical University Joint Institutional Review Board/Ethics Committee (TMU-JIRB) (approval number: N201808042). As this is a retrospective study, the informed consent is not required by TMU-JIRB.

All patients were made to lie in the Jackknife position under spinal or general anesthesia. After anal canal lubrication and insertion of the circular anal dilator, the anvil was inserted, followed by making a single mucosal purse-string suture using 2-0 Prolene at about 2 cm proximal from the dentate line. Different points were anchored based on the degree of the prolapsed mucosa in the DST group. For patients in the PPH group, the stapler was fired after reaching the maximum reinforcement of the staple. After the firing of the stapler, 3-0 vicryl suture ligation and electrocauterization were used for achieving hemostasis. We also removed the external part of hemorrhoid if it existed.

**Figure 2:**
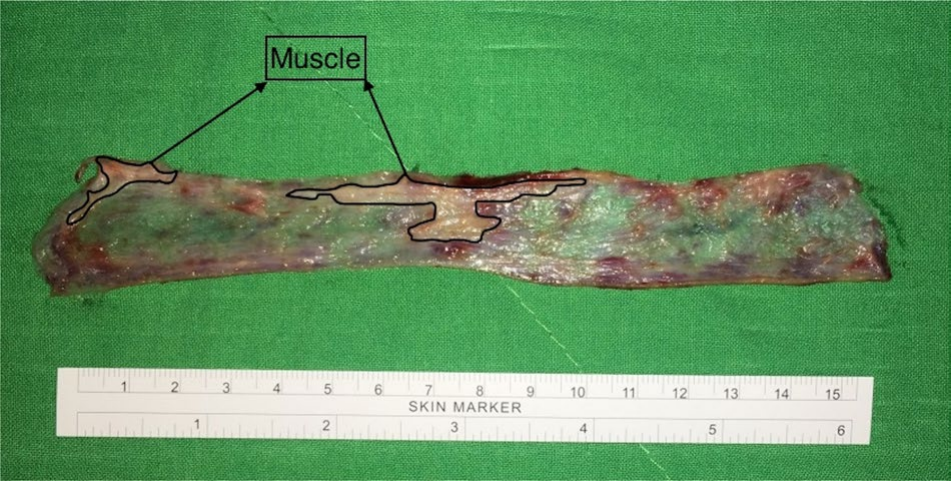
A quantitative histological measurement was made of the muscle contained in each specimen to determine the surface area of mucosa, muscle and muscle.mucosa ratio.

After the surgery, the ring of the excised anorectal mucosa was cut open and flattened on a surgical towel as shown in Fig. 2. The presence of muscle in the specimen was confirmed grossly and photographed. The entire surface area of the mucosa, muscle and the muscle/mucosa ratio were measured by the surgeon using the ImageJ software (National Institutes of Health, Bethesda, MD, USA).

Postoperative analgesia was performed using 40 mg parecoxib injected intravenously every 12 h for the first 24 h, depending on patient request, and 500 mg acetaminophen QID administered orally in the first week. A visual analog scale (VAS) score was used for measuring pain, where 0 represented no pain and 10 represented the worst pain ever experienced. The pain score was recorded by ward nurses on the day of the operation and the first postoperative day. Follow-up in the outpatient clinic was scheduled at 1 week, 3 weeks, and 3 months after surgery. Postoperative bleeding was defined as (1) when the bleeding required surgical intervention or (2) when it warranted hospital admission after patient discharge. Urinary retention was defined as patients requiring urinary catheterization within 7 days after surgery. PPH syndrome was defined as patients complaining about tenesmus, frequency, and fecal urgency in the third month in the postoperative clinic follow-up. The anorectal stricture was defined as patients having difficulty in defecation after the operation and requiring anal dilatation or stricturoplasty in the third month in the postoperative clinic follow-up. Medical records of the patients were used to determine the total number of doses of intravenous analgesic drugs, VAS scores, and postoperative complications such as urinary retention, bleeding, PPH syndrome, and anorectal stricture.

Data were analyzed using the SPSS package (Statistical product and Service Solutions 20.0 for Macintosh; SPSS Inc., Chicago, USA). Data were presented as Mean ± standard deviation. Comparisons were made using the *x*^2^ test or one way analysis of variance (ANOVA) for continuous or categorical variables, respectively, and a p value < 0.05 was considered to be statically significant.

## Results

Table 1 lists the characteristics of the study patients. No significant differences were observed between the two groups in terms of age gender, grade hemorrhoids, and type of symptoms. There was also no difference in the duration of symptoms between the groups, with 78 patients in the DST group and 76 patients in the PPH having had symptoms for more than 1 year.

**Table 1:** Baseline patient characteristic.

	DST	PPH	P value
	n = 100	n = 100	—
Age	50.4 ± 11.1	50.5 ± 10.5	0.93
Gender (Male/Female)	44/56	44/56	1
Hemorrhoid degree (III/IV)	28/72	25/75	0.63
Symptoms (n)			
Pain	42	41	0.89
Bleeding	51	59	0.26
Prolapse	70	63	0.29
Symptoms duration (n)			0.8
<1 month	5	3	
1–3 months	9	12	
3–12months	8	9	
>12months	78	76	

*DST: DST stapler; PPH: PPH stapler.

### Early postoperative results and complications

As shown in Table 2, there were no differences in the operative time, postoperative hospital stay, VAS scores, and analgesic dose between the two groups. The redundant external part of the hemorrhoid was removed after stapler firing in 75 patients in the DST group and 66 patients in the PPH group (*p* = 0.16). Among postoperative complications, there were no differences in urinary retention, early and delayed bleeding, and PPH syndrome (Table 3). All of patients followed at outpatient clinic 3 months after surgery. Five patients in the DST group had the complication of anorectal stricture within 3 months after surgery, whereas none of them in the PPH group had this complication (*p* = 0.02). All these five patients with stricture were readmitted for anal dilatation and stricturoplasty, and none of them developed recurrent strictures.

**Table 2:** Early postoperative results.

	DST	PPH	P value
	n = 100	n = 100	—
Anesthesia (general anesthesia/spinal anesthesia)	2/98	5/95	0.25
Operation Time (min)	13.8 ± 3.1	14.0 ± 4.2	0.64
Excision of external part of hemorrhoid	75	66	0.16
Hospital Stay (day)	1.3 ± 0.4	1.2 ± 0.4	0.08
Analgesic Dose	0.8 ± 0.9	0.7 ± 1.0	0.52
Pain score			
OP day	2.7 ± 1.7	2.4 ± 1.7	0.89
POD1	1.9 ± 1.2	1.8 ± 1.5	0.26

*OP: operation; POD: Postoperative Day.

*DST: DST stapler; PPH: PPH stapler.

**Table 3:** Complications.

	DST	PPH	P value
	n = 100	n = 100	—
Early			
Urinary retention	18	12	0.24
Bleeding (<24 hours)	1	0	0.63
Bleeding (>24 hours)	1	1	1
Late			
Stricture	5	0	**0**.**02**
PPH syndrome	0	2	0.16

*DST: DST stapler; PPH: PPH stapler.

### Assessment of the anorectal mucosa ring

Anorectal mucosal specimens were analyzed, and the results are shown in Table 4. The mean surface area of the specimen in the DST group was 37.2 ± 6.5 cm^2^, whereas it was 34.8 ± 8.4 cm^2^ in the PPH group (*p* = 0.03). The mean muscle/mucosa ratio was 0.048 in the DST group compared to 0.024 in the PPH group (*p* = 0.04). The mean surface area of the muscle was 1.5 ± 3.2 cm^2^ in the DST group versus 0.7 ± 1.6 cm^2^ in the PPH group (*p* = 0.02). Moreover, data of the five patients with anorectal stricture were analyzed and compared to the data of the remaining 95 patients without anorectal stricture in the DST group. Patients with postoperative anorectal stricture were of younger age (*p* = 0.002) and had more surface area of the muscle (*p* = 0.03) and a higher muscle/mucosa ratio (*p* = 0.03) than patients who did not have stricture. These results are presented in Table 5.

**Table 4:** Operative Specimen analysis.

	DST	PPH	P value
	n = 100	n = 100	—
Surface Area (cm^2^)	37.2 ± 6.5	34.8 ± 8.4	**0**.**03**
Muscle involved (n)	29	25	0.52
Muscle/Mucosa Ratio	0.05 ± 0.10	0.02 ± 0.06	**0**.**04**
Surface Area of Muscle (cm^2^)	1.5 ± 3.2	0.7 ± 1.6	**0**.**02**

*DST: DST stapler; PPH: PPH stapler.

**Table 5:** Analysis of patients in DST group.

	No stricture	Stricture	P value
	n = 95	n = 5	—
Age	51.2 ± 10.8	35.4 ± 5.9	**0**.**002**
Gender	42/53	2/3	0.85
Hemorrhoid degree (III/IV)	26/69	2/3	0.54
Surface Area (cm^2^)	37.3 ± 6.6	34.9 ± 4.2	0.43
Muscle/Mucosa Ratio	0.04 ± 0.09	0.14 ± 0.21	**0**.**03**
Surface Area of Muscle (cm^2^)	1.4 ± 2.9	4.5 ± 6.4	**0**.**03**

## Discussion

The principle of SH involves performing circular mucosectomy to achieve anal lifting and avoid irritation to the sensitive anoderm^15^. Thus, SH is a less painful procedure for patients as it avoids cutaneous wounds and also allows them to return to their normal activities in a shorter time. According to recent studies, although SH tends to result in a higher recurrence rate^13,16^, the complication rate of hemorrhoidopexy is not inferior to that of conventional hemorrhoidectomy in terms of bleeding, pain, pruritus, or fecal urgency^17^. In the present study, both devices were found to be feasible and safe for performing SH. The operative time, duration of hospital stay, VAS scores, and analgesic doses showed no statistically significant differences between the two groups.

Postoperative hemorrhage is a relatively common complication in SH, occurring in 1.5–15.6% of the patients^18–21^, and the maximum bleeding occur**s** immediately after surgery due to inadequate hemostasis. In SH, the closed staple height that determines the compression is 0.75 mm in PPH stapler and 1.5 mm in DST stapler. Literature reports that although the DST stapler is higher than that in the PPH stapler, the postoperative bleeding rate is not inferior. Kam *et al*. reviewed 1118 patients who underwent SH with DST stapler and found that 4.6% of the patients had postoperative bleeding that required readmission^22^. Ng *et al*. also reviewed 3711 patients using the PPH stapler and 4.3% of them had postoperative bleeding^19^. Using the DST stapler resulted in better intraoperative hemostasis than the PPH stapler in a randomized clinical trial, but the rate of postoperative bleeding was not statistically different^14^. In our practice, electrocauterization or sutures on the bleeders at the staple line were used for achieving hemostasis, which resulted in only one patient in the DST group with early postoperative bleeding in this study.

The surface area of the excised mucosa in SH is an indicator for the surgeon to determine how much of the prolapsed mucosa has been lifted. Giuratrabocchetta *et al*. reported that the DST stapler can resect a significantly larger area of mucosa than the PPH stapler due to the wider capacity of its shaft case^14^, and a similar result was found in the present study. Although recurrence prolapse is more likely to be due to remove inadequate volume of prolapse mucosa^23^, there is no difference in recurrence rate between DST and PPH stapler in a long-term randomized control trial^24^.

One of the serious complications after hemorrhoidal surgery is anorectal stricture. Anorectal stricture generally occurs within 3 months after surgery in SH^19,25^; its incidence ranges from 0% to 5%^11,25,26^ and is similar compared to that with conventional hemorrhoidectomy among current studies^25^. In the conventional hemorrhoidectomy, the pathophysiology of the anal stricture is removal of large areas of the anoderm and the hemorrhoidal rectal mucosa, without sparing adequate mucocutaneous bridges, leading to scarring and a progressive chronic stricture^25,27^. However, SH generally causes rectal strictures whose pathophysiology has not yet been clearly understood. The potential mechanism responsible for causing a stricture is ring dehiscence followed by submucosal inflammation, and another theory is that the stapled ring is too close in the anal canal and that the squamous skin cells react by scarring and shrinking^19,22,27^. In this study, all the five patients with anorectal stricture had a resected muscularis propria of lower rectum in the specimens and the muscle/mucosa ratio and the surface area of the resected muscle were significantly larger than those of patients without stricture. The fibrous tissue with metal staples was noted at the stricture ring proximal to the dentate line in all the five patients during anal dilatation and stricturoplasty. Accordingly, we proposed that lots of muscle resection in lower rectum induced metal staples embedded in rectal muscle and subsequently inflammation reaction therein and finally anorectal stricture.

Deep or too much muscle cutting in SH also induced a few serious complications such as rectal perforation, pelvic abscess, rectovaginal fistula, and intra-abdominal bleeding^28–31^. Therefore, an important step in SH is to excise a sufficient area of the rectal mucosa to achieve the ideal anal lifting level by avoiding muscle involvement. We should always be aware of the anatomic layer within the anal canal so that we could include only the mucosa and the submucosal layer while making the purse-string suture to avoid sphincter muscle damage and then decrease the complications. In our practice, the same purse-string suture was performed in patients in both groups; however, a higher muscle: mucosa ratio and more muscle resection were observed in the DST group. For a better understanding of the mechanisms causing different muscle resections between the two groups, it is necessary to discuss the procedure from several different aspects. Although the diameter in both staplers was 33 mm, the structure and designs were different between these two devices. First, the PPH stapler has a thicker anvil as well as a plastic ring in the case, due to which the capacity for tissue resection after case closure might be less than that of the DST stapler. This might result in more tissue resection by the DST stapler. Second, regarding the utilization of the DST stapler, there are only three anchor points to choose. Therefore, there is no sufficient amount of anchor points to make precise adjustment according to the conditions of different patients. These two reasons are possibly responsible for the differences in the muscle/mucosa ratio and the surface area of the resected muscle, which also resulted in the anorectal stricture in the DST group. Although our results tend to indicate a higher anorectal stenosis rate using the DST stapler, there are a few potential advantages of it that we cannot overlook. The trial that we had mentioned earlier also demonstrated a significantly better hemostatic performance^14^. In another case series, Pramateftakis *et al*. indicated better modulation and inspection using the DST stapler due to the detachable anvil^32^.

Younger age was a predictor of a higher postoperative anorectal stricture rate in the DST group. Young patients have less muscle atrophy or less mucosal prolapse, which contributes to the resection of more muscle and a higher anorectal stricture rate. Although we found a statistical significance between the resected muscle area and the stricture, there were several patients who underwent muscle resection but did not experience symptoms of anorectal stricture. Therefore, further research is required to understand the correlation between muscle resection and anorectal stricture.

Higher recurrence rate after SH than conventional hemorrhoidectomy is a major concern after postoperative long-term following^12,33^. However the prolapse recurrence rate varies widely in different studies. In a 12-year follow-up study, the recurrence rate was 40.9% after SH^34^, and in another long-term follow-up study, SH and conventional hemorrhoidectomy have similar rate of additional medical or surgical treatment for recurrence^35^. Many studies have pointed out that patients with grade 2 to 3 hemorrhoidal disease without external prolapsed skin have satisfied therapeutic effect after SH^36–39^. Kim, *et al*. reported that the recurrence rate is similar in SH and conventional hemorrhoidectomy under appropriate patient selection and resection of excess external prolapsed skin if necessary^38^. In our study, 75% of patients in DST group and 66% in PPH group received excision of external part of hemorrhoid, and no recurrence occurred in all of patients within 3 months following.

This study still has some limitations. First, this was a single-surgeon retrospective cohort matched study, which makes some collection bias inevitable and lacks of generalizability. However, all the study patients underwent surgery by the same surgeon and were followed up at the same institution using the same routine, which could have substantially decreased the bias. Second, the selections between different devices were not randomized, but based on the available storage at the admission time. However, we treated the patient circumstantially with standard protocol. There was no additional referral or different practice among the whole treatment course. Third, this study lacks long-term follow up to evaluate some concerning results, i.e., the recurrence of mucosal prolapse, chronic pain or fecal urgency. Fourth, about the utilization of DST stapler, the anchor of each patient was not recorded, and the relationship between the surface area of muscle/mucosa and level of anchor point cannot be analyzed in this study. Finally, there might be differences in the hemostasis ability of the two devices, but we were not able to find any statistical difference as we used stay suture and electrocauterization for achieving hemostasis in all patients. Immediate evaluation for bleeding right after stapler firing might have depicted the difference more clearly.

Recently, a new dedicated and high volume device for SH, TST STARR plus (TST Starr plus, Touchstone International Medical Science Co, Ltd, Suzhou, China), had developed. This device could reduce the recurrence rate through the possibility of more tissue resection basis of the entity of the prolapse^23^. Naldini, *et al*. reported that 1.9% prolapsed recurrence after operation in a median follow-up of 14.5 months^40^. This series also showed no occurrence of anorectal stricture and it could be due to the larger diameter of stapler (36 mm) than PPH (33 mm) or DST (33 mm). The surgical outcomes of this new generation device should be evaluated by a comparison between old stapler devices.

In conclusion, the short-term outcomes of stapled hemorrhoidopexy were almost similar when using either the DST or the PPH stapler, except that the surface area of the resected mucosa was larger in the DST group. Besides, the surface area of the resected muscle and the muscle/mucosa ratio were larger in the DST group than in the PPH group. Patients in the DST group had a tendency to develop postoperative stricture. Further study is required to understand the correlation between muscle resection and stricture.
